# Development and temporal validation of a prediction model for cognitive impairment in older adults with hearing loss based on the population health risk management framework

**DOI:** 10.3389/fpubh.2026.1873714

**Published:** 2026-07-16

**Authors:** Xianyan Xu, Mengting Li, Xuling Gao, Yan Wang, Jun Ge, Rong Zhao, Li Ma, Qiankun Liu

**Affiliations:** 1 Department of Nursing, Bengbu First People’s Hospital, Bengbu, Anhui, China; 2 School of Nursing, Bengbu Medical University, Bengbu, Anhui, China

**Keywords:** cognitive impairment, older adults, hearing loss, machine learning, risk prediction

## Abstract

**Background:**

Older adults with hearing loss are at increased risk of cognitive impairment, yet tailored risk stratification tools remain limited.

**Methods:**

In this cross-sectional study, 524 adults aged ≥60 years with hearing loss were enrolled between June 2023 and September 2024. Participants were divided by enrollment period into a development cohort (June 2023 to May 2024, *n* = 367) and a temporally independent validation cohort (June 2024 to September 2024, *n* = 157). Cognitive function was assessed using the Montreal Cognitive Assessment, with a score <26 indicating cognitive impairment after correction for education. Candidate predictors were selected based on the Integrated Framework for Population Health Risk Management. Feature selection was performed using LASSO regression, and six machine-learning models were developed and compared. Model performance was evaluated using AUC, F1 score, sensitivity, specificity, Youden index, PPV, NPV, calibration curves, and decision curve analysis. The final candidate model was optimized using grid search with five-fold cross-validation, and SHAP was applied for interpretability.

**Results:**

The prevalence of cognitive impairment was 40.8%. LASSO identified five key predictors: age, pure-tone average, depression, hearing-aid use, and social activities. Multivariable analysis showed that depression, older age, and higher pure-tone average were associated with a higher likelihood of cognitive impairment, whereas hearing-aid use and participation in social activities were protective factors. Among the six models, Random Forest showed the best overall performance in the validation cohort. After hyperparameter tuning, the optimized Random Forest model achieved an AUC of 0.952 in the training cohort and 0.871 in the validation cohort. In the validation cohort, the F1 score, sensitivity, Youden index, and NPV increased to 0.737, 0.779, 0.583, and 0.863, respectively. SHAP analysis indicated that pure-tone average, age, and social activities were the most influential predictors in the optimized model.

**Conclusion:**

The optimized five-variable Random Forest model demonstrated good predictive performance for cognitive impairment in older adults with hearing loss. This low-cost and interpretable tool may support early screening, risk stratification, and targeted intervention in clinical and community settings.

## 1 Introduction

With the rapid acceleration of global population aging, hearing loss has become one of the most common sensory impairments among older adults, and its prevalence continues to increase with advancing age (1). The Global Burden of Disease study reports that more than 1.5 billion people worldwide are affected by varying degrees of hearing loss, including approximately 403 million with moderate or worse impairment (2). Hearing loss not only compromises communication ability in older adults, but may also lead to restricted social interaction, emotional disorders, and reduced quality of life (3). Our previous studies have shown that hearing loss is closely associated with cognitive decline and may increase the risk of cognitive impairment through pathways such as social isolation and depression (4–7). Meanwhile, cognitive impairment—particularly mild cognitive impairment (MCI) and dementia—has become a major public health concern in the context of global aging. As a critical transitional stage from normal cognition to dementia, MCI has substantial value for early identification and intervention (8, 9). In older populations, hearing loss frequently coexists with cognitive impairment; however, communication difficulties and related psychosocial problems caused by hearing decline can contribute to under-recognition or misdiagnosis of cognitive impairment (10). Therefore, early screening and intervention for cognitive impairment among older adults with hearing loss are of great importance for delaying cognitive deterioration, improving prognosis, and enhancing quality of life.

In recent years, an increasing number of studies have explored factors associated with cognitive impairment among older adults with hearing loss. Available evidence suggests that its onset and progression are driven by multiple interacting determinants, primarily involving physiological and psychological factors, environmental and occupational exposures, and social and behavioral influences. Age-related auditory degeneration and chronic conditions such as hypertension, diabetes, and cardiovascular disease may contribute to cognitive decline by affecting auditory input, cerebral blood flow, and neural function (11–13). Psychological problems including social isolation, depression, and anxiety can further increase cognitive burden and accelerate cognitive deterioration (14). Long-term noise exposure, adverse living environments, and high-stress occupational characteristics may also impair cognitive function (15). Conversely, adequate social support, healthy lifestyles, active cognitive engagement, and timely use of hearing aids and hearing rehabilitation may help delay cognitive decline (16–18). Although research on these determinants has made initial progress, most studies have focused on single domains (eg, demographic, psychological, or physiological factors) and lack integration under a systematic theoretical framework. To more comprehensively identify and organize relevant factors, this study introduces the Integrated Framework for Population Health Risk Management (IFPHRM) proposed by Krewski (19). This framework emphasizes the systematic assessment of health problems from both “health management” and “risk management” perspectives and categorizes risk determinants into three levels: physiological and psychological, environmental and occupational, and social and behavioral factors. It provides theoretical support for health risk identification and prioritization of interventions among older adults with hearing loss who are at risk of cognitive impairment.

With the development of artificial intelligence and big data technologies, disease prediction models constructed using different data sources and algorithms have become an important approach to improving the efficiency of early identification and screening for cognitive impairment. Existing studies have developed various prediction models for cognitive impairment in populations such as disabled older adults and patients with hypertension or diabetes, demonstrating certain discriminative ability (20–22). However, most models still suffer from limitations including insufficiently systematic inclusion of predictors, inadequate external validation, and limited clinical generalizability and applicability (23). In addition, current research has primarily focused on the general older population, patients with chronic diseases, or individuals with brain injury, whereas prediction models targeting older adults with hearing loss—a high-risk group for cognitive impairment—remain scarce, thereby limiting the applicability of existing models in this population. Our previous work also found that many related studies rely on questionnaire-based public databases and lack objective audiometric data, which may introduce sample bias. Therefore, guided by the IFPHRM framework, this study aims to systematically integrate multidimensional factors across physiological and psychological, environmental and occupational, and social and behavioral domains. Using more objective hearing assessment data, we will develop and validate a machine learning–based risk prediction model for cognitive impairment in older adults with hearing loss, to enhance early identification and risk-stratified management in this high-risk population.

## 2 Methods

### 2.1 Study participants

This was a cross-sectional study. From June 2023 to September 2024, a total of 524 older adults with hearing impairment were consecutively recruited from the otolaryngology outpatient clinic of Bengbu First People’s Hospital (the designated hospital for hearing disability assessment in Bengbu) and the Bengbu community disability assessment center. Participants enrolled from June 2023 to May 2024 were assigned to the model development cohort (*n* = 367), whereas those enrolled from June 2024 to September 2024 were assigned to the external validation cohort (*n* = 157).

1. Inclusion criteria: ① Age ≥ 60 years; ② According to the WHO World Report on Hearing, hearing impairment was defined as a pure-tone average (PTA) in the better ear at 0.5, 1, 2, and 4 kHz of ≥ 20 dB HL (24); ③ No history of psychotropic medication use; ④ The participant and accompanying person provided informed consent to participate in this study.
2. Exclusion criteria: ① History of neurological diseases, including Alzheimer’s disease, mixed dementia, epilepsy, Parkinson’s disease, or multiple sclerosis, that precluded effective completion of questionnaires or interviews.

### 2.2 Variable selection

Based on the theoretical framework of the IFPHRM, which emphasizes a multidimensional assessment of health determinants across physiological, psychological, environmental, and social-behavioral domains, this study developed a systematic and theory-driven set of variables focusing on factors related to older adults with hearing loss. Candidate predictors were selected through expert consultation (neurology, nursing, and statistics) and a comprehensive literature review, and were finalized accordingly. All variables were classified into three categories: (1) physiological and psychological factors, including physiological health indicators such as age, sex, diabetes, hypertension, and number of chronic conditions, as well as psychological health indicators such as anxiety, depression, and fatigue; (2) environmental and occupational factors, including place of residence (urban/rural) and exposure to stress; and (3) social and behavioral factors, including educational level, living status, income level, smoking and alcohol use, exercise frequency, hearing aid use, and multidimensional social participation (productive activities, cultural/spiritual activities, physical activities, and social activities).

### 2.3 Variables and measurement methods

#### 2.3.1 Hearing assessment

Pure-tone audiometry was performed using a professional audiometer (Audiometer 1,081) equipped with matching headphones (ME-70) and a bone vibrator (B-71). All hearing assessments were conducted in a standardized hospital sound-treated audiometric room, with sound insulation greater than 50 dB, under the guidance of certified audiologists. Air-conduction hearing thresholds were measured at 500, 1000, 2000, and 4,000 Hz, and the average of these four thresholds was calculated as the pure-tone average (PTA).

#### 2.3.2 Cognitive function

Cognitive function was assessed using the Montreal Cognitive Assessment (MoCA). The MoCA evaluates multiple cognitive domains, including attention, executive function, memory, language, visuospatial ability, abstraction, calculation, and orientation, with total scores ranging from 0 to 30; higher scores indicate better cognitive performance. A cutoff score of <26 was used to define cognitive impairment, which has been widely adopted in previous studies (25). For participants with ≤12 years of education, one additional point was added to the total score to correct for educational effects. The MoCA has demonstrated good reliability and validity among older populations. In this study, the Cronbach’s *α* of the MoCA was 0.838.

#### 2.3.3 General sociodemographic questionnaire

This questionnaire was developed under the guidance of neurology specialists, nursing experts, and statisticians, and it comprises variables including physiological and psychological factors (age, sex, diabetes, hypertension, number of chronic diseases, and fatigue), environmental and occupational factors (place of residence and stress), and social and behavioral factors (educational level, living arrangement, smoking, alcohol consumption, income, exercise frequency, mobile phone use, hearing-aid use, participation in productive activities—such as paid work, unpaid volunteer service, and caregiving for family members—participation in mental and cultural activities—such as reading, gardening, using the internet, traveling, and shopping—participation in physical activities—such as walking, dancing/fitness, and housework—and participation in social activities—such as visiting relatives and friends, attending senior universities, and participating in clubs and chess/card games).

#### 2.3.4 Anxiety

In this study, the Self-Rating Anxiety Scale (SAS) was used to assess anxiety levels among older adults with hearing loss. The SAS consists of 20 items rated on a 4-point Likert scale (1–4), with items 5, 9, 13, 17, and 19 scored in reverse (26). The sum of item scores yields a raw score, and the standard score (0–100) is calculated as the raw score × 1.25 and rounded to the nearest integer. A standard score ≥50 indicates the presence of anxiety symptoms. In this study, the scale demonstrated a Cronbach’s *α* of 0.731.

#### 2.3.5 Depression

In this study, the 10-item Center for Epidemiologic Studies Depression Scale (CESD-10) was used to assess depressive symptoms (27). The scale evaluates participants’ emotional and behavioral manifestations over the past week and consists of 10 items rated on a 4-point scale (0–3), yielding a total score ranging from 0 to 30. The items “I am hopeful about the future” and “I am happy” are reverse scored. A total score ≥10 was used as the cutoff value for identifying depressive symptoms (28). In this study, the scale demonstrated good reliability and validity (Cronbach’s *α* = 0.815).

### 2.4 Quality control

(1) Before the study began, the research team contacted the heads of relevant hospital departments and the audiometrists to clarify the purpose of the survey and obtain their support. (2) Prior to distributing the questionnaires, a survey team was established. All investigators received standardized training to ensure a full understanding of the study objectives, questionnaire content, survey procedures, and precautions. Investigators were required to be familiar with each dimension of the questionnaire scales and able to interpret hearing-related data. The completion time was controlled at approximately 15–20 min. Mock surveys were organized, and any problems encountered during the simulation were addressed promptly. After the training, team members were assessed on the training content, and only those who passed were allowed to participate in the formal survey. (3) Quality control procedures were implemented for the questionnaires and scales, and each returned questionnaire was checked to ensure completeness. If any item was missing, participants were asked to provide the missing information to ensure that all questions were fully answered before submission. (4) During the survey, issues were summarized regularly and handled in a timely manner. The team leader was responsible for collecting and reviewing the questionnaires, which were independently double-checked by two staff members; invalid questionnaires were excluded to ensure the authenticity and accuracy of the data.

### 2.5 Statistical analysis

Statistical analysis and model development were conducted using SPSS version 26.0, R version 4.5.2, and Python version 3.12.7. Categorical variables are presented as counts and percentages, and between-group comparisons were performed using the *χ*^2^ test. Continuous variables with a normal distribution are presented as mean ± standard deviation and were compared using the independent-samples t-test; otherwise, they were presented as median and interquartile range and compared using nonparametric tests. In the modeling set, variables with *p* < 0.05 in univariate analysis were entered into least absolute shrinkage and selection operator (LASSO) regression for feature selection. Cross-validation was used to determine the optimal penalty parameter (*λ*) (29, 30), and variables with non-zero coefficients were retained. Multicollinearity among the selected variables was assessed using the variance inflation factor (VIF). Based on the retained predictors, six machine learning models were developed, including Logistic regression, Decision Tree, Naive Bayes, Random Forest, Support Vector Machine, and XGBoost. Model performance was evaluated using receiver operating characteristic curves generated by Bootstrap resampling with 1,000 iterations. The models were compared in the training and validation sets using AUC, F1 score, sensitivity, specificity, Youden index, positive predictive value (PPV), and negative predictive value (NPV). Calibration curves and decision curve analysis were further used to assess model calibration and clinical net benefit. The model with the best overall performance in the validation set was selected as the final candidate model. Subsequently, grid search combined with five-fold cross-validation was used in the training set to optimize the hyperparameters of the final candidate model. The optimized model was then re-evaluated in the validation set using the same performance metrics. Finally, SHAP analysis was applied to the optimized final model to interpret feature contributions and generate beeswarm plots. All tests were two-sided, and *p* < 0.05 was considered statistically significant.

### 2.6 Ethical considerations

This study was approved by the Ethics Committee of the First People’s Hospital of Bengbu (Approval no. BBYYLWPJ2025022) and by the ethics committee of Bengbu Medical University (Approval no. 2025-179). All participants signed written informed consent prior to participation, ensuring that their involvement was voluntary and that they were fully informed of the study objectives and relevant procedures.

## 3 Results

### 3.1 Baseline characteristics of older adults with hearing impairment in the modeling and validation sets

A total of 524 older adults with hearing impairment were included, comprising 367 participants in the modeling set and 157 in the validation set. Comparisons of baseline characteristics between the two sets showed that the educational level was higher in the modeling set, whereas social participation was higher in the validation set; both differences were statistically significant (all *p* < 0.001). No statistically significant differences were observed for the remaining variables (all *p* > 0.05). Overall, the baseline data of the modeling and validation sets were comparable, except for educational level and social participation (Table 1).

**Table 1:** General characteristics of older adults with hearing impairment in the modeling and validation sets.

Variable	Category	Validation set (*n* = 157)	Modeling set (*n* = 367)	*X*^2^/Z	*p* value
Education	Illiterate	56 (35.67%)	139 (37.87%)	19.121	<0.001
	≤6 years	84 (53.50%)	134 (36.51%)		
	≥6 years	17 (10.83%)	94 (25.61%)		
Sex	Female	72 (45.86%)	162 (44.14%)	0.071	0.790
	Male	85 (54.14%)	205 (55.86%)		
Monthly income	<2000 CNY	56 (35.67%)	119 (32.43%)	0.846	0.655
	2000–5,000 CNY	83 (52.87%)	210 (57.22%)		
	>5,000 CNY	18 (11.46%)	38 (10.35%)		
Residence	Rural	56 (35.67%)	115 (31.34%)	0.753	0.386
	Urban	101 (64.33%)	252 (68.66%)		
Living arrangement	Living alone	63 (40.13%)	152 (41.42%)	0.032	0.859
	Not living alone	94 (59.87%)	215 (58.58%)		
Smoking	No	89 (56.69%)	209 (56.95%)	0.001	0.999
	Yes	68 (43.31%)	158 (43.05%)		
Alcohol consumption	No	108 (68.79%)	261 (71.12%)	0.185	0.667
	Yes	49 (31.21%)	106 (28.88%)		
Diabetes	No	74 (47.13%)	185 (50.41%)	0.35	0.554
	Yes	83 (52.87%)	182 (49.59%)		
Hypertension	No	85 (54.14%)	195 (53.13%)	0.013	0.908
	Yes	72 (45.86%)	172 (46.87%)		
Hyperlipidemia	No	118 (75.16%)	247 (67.30%)	2.85	0.091
	Yes	39 (24.84%)	120 (32.70%)		
Number of chronic diseases	0	39 (24.84%)	81 (22.07%)	0.938	0.626
	1	55 (35.03%)	144 (39.24%)		
	≥2	63 (40.13%)	142 (38.69%)		
Hearing aid	No	89 (56.69%)	213 (58.04%)	4.854	0.088
	Occasionally	43 (27.39%)	119 (32.43%)		
	Frequently	25 (15.92%)	35 (9.54%)		
Exercise frequency	None	71 (45.22%)	167 (45.50%)	0.001	0.999
	≥Once per week	86 (54.78%)	200 (54.50%)		
Productive activities	No	106 (67.52%)	251 (68.39%)	0.009	0.924
	Yes	51 (32.48%)	116 (31.61%)		
Mental activities	No	82 (52.23%)	189 (51.50%)	0.003	0.954
	Yes	75 (47.77%)	178 (48.50%)		
Physical activities	No	83 (52.87%)	194 (52.86%)	0.001	0.999
	Yes	74 (47.13%)	173 (47.14%)		
Social activities	No	83 (52.87%)	263 (71.66%)	16.491	<0.001
	Yes	74 (47.13%)	104 (28.34%)		
Depression	No	73 (46.50%)	165 (44.96%)	0.052	0.820
	Yes	84 (53.50%)	202 (55.04%)		
Stress	Low	39 (24.84%)	84 (22.89%)	0.268	0.875
	Moderate	65 (41.40%)	153 (41.69%)		
	High	53 (33.76%)	130 (35.42%)		
Fatigue	No	66 (42.04%)	148 (40.33%)	0.072	0.789
	Yes	91 (57.96%)	219 (59.67%)		
PTA		69.0 (48.0,83.0)	63.0 (43.0,82.0)	1.603	0.109
Age		69.0 (63.0,79.0)	70.0 (64.0,79.0)	−0.345	0.730
Loneliness		15.0 (12.0,24.0)	15.0 (11.0,22.0)	−0.166	0.868
Anxiety		44.0 (26.0,55.0)	40.0 (26.0,50.0)	1.079	0.280
Social support		45.0 (29.0,60.0)	40.0 (27.0,56.5)	1.660	0.097

### 3.2 Univariate analysis of cognitive impairment in older adults with hearing loss

As shown in Table 2, significant differences were observed between the cognitive impairment group and the normal group in education level, living arrangement, alcohol consumption, diabetes, hypertension, number of chronic diseases, hearing aid use, exercise frequency, participation in mental activities, participation in physical activities, social activities, depression, stress, PTA, age, loneliness, anxiety, and social support (all *p* < 0.05). In contrast, sex, monthly income, place of residence, smoking, hyperlipidemia, and participation in productive activities were not significantly different between the two groups (all *p* > 0.05). All variables with statistical significance in the univariate analysis were subsequently entered into the multivariable logistic regression model.

**Table 2:** Univariate analysis of cognitive impairment in older adults with hearing impairment.

Variable	Category	Normal (*n* = 219)	Cognitive impairment (*n* = 148)	X^2^/Z	*p* value
Education	Illiterate	57 (26.03%)	79 (53.38%)	29.794	<0.001
	≤6 years	132 (60.27%)	61 (41.22%)		
	≥6 years	30 (13.70%)	8 (5.41%)		
Sex	Female	106 (48.40%)	56 (37.84%)	3.58	0.059
	Male	113 (51.60%)	92 (62.16%)		
Monthly income	<2000 CNY	63 (28.77%)	56 (37.84%)	3.472	0.176
	2000–5,000 CNY	131 (59.82%)	79 (53.38%)		
	>5,000 CNY	25 (11.42%)	13 (8.78%)		
Place of residence	Rural	63 (28.77%)	52 (35.14%)	1.382	0.240
	Urban	156 (71.23%)	96 (64.86%)		
Living arrangement	Living alone	81 (36.99%)	71 (47.97%)	3.952	0.047
	Not living alone	138 (63.01%)	77 (52.03%)		
Smoking	No	134 (61.19%)	75 (50.68%)	3.563	0.059
	Yes	85 (38.81%)	73 (49.32%)		
Alcohol consumption	No	170 (77.63%)	91 (61.49%)	10.427	0.001
	Yes	49 (22.37%)	57 (38.51%)		
Diabetes	No	126 (57.53%)	59 (39.86%)	10.334	0.001
	Yes	93 (42.47%)	89 (60.14%)		
Hypertension	No	130 (59.36%)	65 (43.92%)	7.848	0.005
	Yes	89 (40.64%)	83 (56.08%)		
Hyperlipidemia	No	154 (70.32%)	93 (62.84%)	1.919	0.166
	Yes	65 (29.68%)	55 (37.16%)		
Number of chronic diseases	0	61 (27.85%)	20 (13.51%)	19.565	<0.001
	1	92 (42.01%)	52 (35.14%)		
	≥2	66 (30.14%)	76 (51.35%)		
Hearing aid	No	84 (38.36%)	101 (68.24%)	35.642	<0.001
	Occasionally	36 (16.44%)	5 (3.38%)		
	Frequently	99 (45.21%)	42 (28.38%)		
Exercise frequency	None	78 (35.62%)	89 (60.14%)	20.433	<0.001
	≥Once per week	141 (64.38%)	59 (39.86%)		
Participation in productive activities	No	153 (69.86%)	98 (66.22%)	0.388	0.534
	Yes	66 (30.14%)	50 (33.78%)		
Participation in mental activities	No	80 (36.53%)	109 (73.65%)	47.242	<0.001
	Yes	139 (63.47%)	39 (26.35%)		
Participation in physical activities	No	78 (35.62%)	116 (78.38%)	63.105	<0.001
	Yes	141 (64.38%)	32 (21.62%)		
Social activities	No	89 (40.64%)	129 (87.16%)	77.344	<0.001
	Yes	130 (59.36%)	19 (12.84%)		
Depression	No	132 (60.27%)	33 (22.30%)	49.949	<0.001
	Yes	87 (39.73%)	115 (77.70%)		
Stress	Low	64 (29.22%)	20 (13.51%)	13.394	0.001
	Mid	88 (40.18%)	65 (43.92%)		
	High	67 (30.59%)	63 (42.57%)		
PTA		54.0(40.0,69.0)	80.5 (74.0,90.0)	−10.167	<0.001
Age		66.0 (62.0,72.0)	78.0 (72.0,82.0)	−9.142	<0.001
Loneliness		13.0 (9.0,19.0)	22.0 (15.0,25.0)	−7.005	<0.001
Anxiety		26.0 (24.0,44.0)	48.0 (43.0,56.0)	−9.062	<0.001
Social support		44.0 (32.0,60.0)	29.0 (24.0,44.0)	5.989	<0.001

### 3.3 Feature selection of predictors

Variables that were statistically significant in the univariate analysis (*p* < 0.05) were further entered into LASSO regression for feature selection. After determining the optimal penalty parameter (*λ*) via cross-validation, five core predictors with non-zero coefficients were ultimately retained: age, PTA, hearing aid use, social activities, and depression.

### 3.4 Multivariable analysis of cognitive impairment in older adults with hearing loss

**Figure 1:**
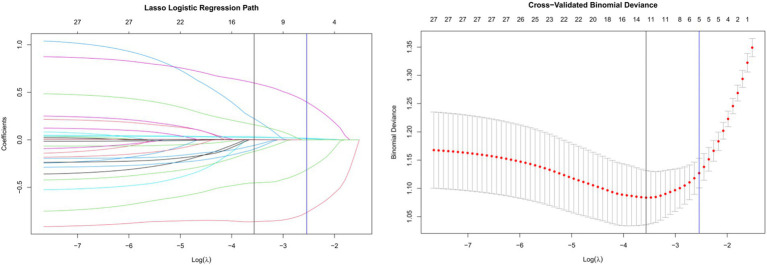
LASSO regression for feature selection in the prediction model of cognitive impairment in older adults with hearing loss.

The five variables selected by LASSO regression were entered into a multivariable logistic regression analysis. As shown in Table 3, depression, age, hearing-aid use, social activities, and PTA were independently associated with cognitive impairment in older adults with hearing loss. Specifically, participants with depression were more likely to have cognitive impairment than those without depression (OR = 2.565, 95% CI: 1.342–4.903, *p* = 0.004). Increasing age was associated with a higher likelihood of cognitive impairment (OR = 2.146, 95% CI: 1.574–2.924, *p* < 0.001), and higher PTA was also associated with an increased likelihood of cognitive impairment (OR = 2.581, 95% CI: 1.821–3.657, *p* < 0.001). Compared with participants who did not use hearing aids, those who used hearing aids sometimes (OR = 0.238, 95% CI: 0.067–0.846, *p* = 0.027) or frequently (OR = 0.437, 95% CI: 0.236–0.810, *p* = 0.009) had a lower likelihood of cognitive impairment. In addition, participation in social activities was identified as a protective factor against cognitive impairment (OR = 0.297, 95% CI: 0.150–0.589, *p* = 0.001; Figure 1).

**Table 3:** Multivariable analysis of cognitive impairment in older adults with hearing loss.

Predictors	*β*	SE	Odds ratio	95% CI	*p* value
Depression (Reference NO)					
Yes	0.942	0.331	2.565	1.342–4.903	0.004
Age					
	0.763	0.158	2.146	1.574–2.924	<0.001
Hearing aid (Reference group: NO)					
Sometimes	−1.434	0.647	0.238	0.067–0.846	0.027
Frequently	−0.872	0.314	0.437	0.236–0.810	0.009
Social activities (Reference group: NO)					
Yes	−1.213	0.349	0.297	0.150–0.589	0.001
PTA					
	0.948	0.178	2.581	1.821–3.657	<0.001

### 3.5 Construction and evaluation of prediction models for cognitive impairment in older adults with hearing loss

**Figure 2:**
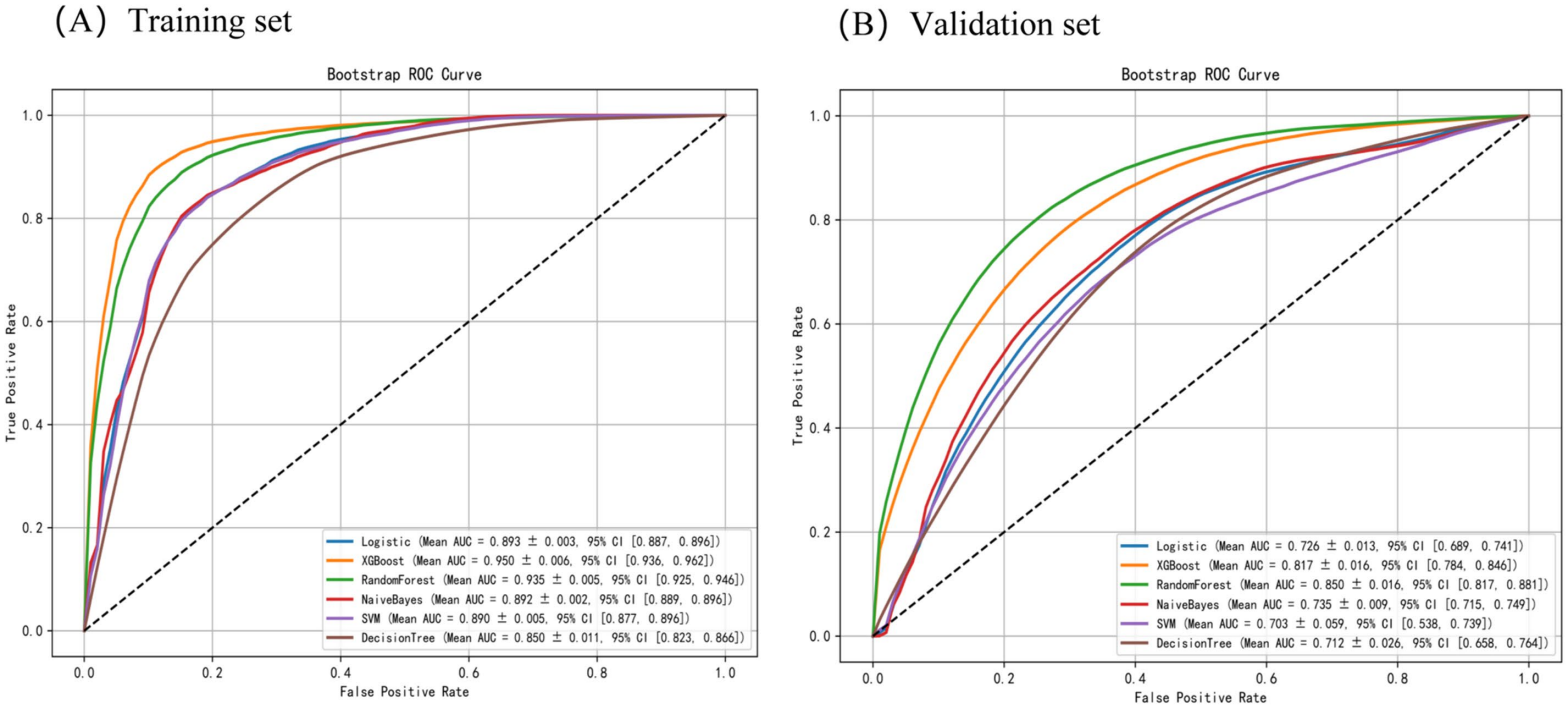
ROC curves of prediction models for cognitive impairment in older adults with hearing loss.

**Figure 3:**
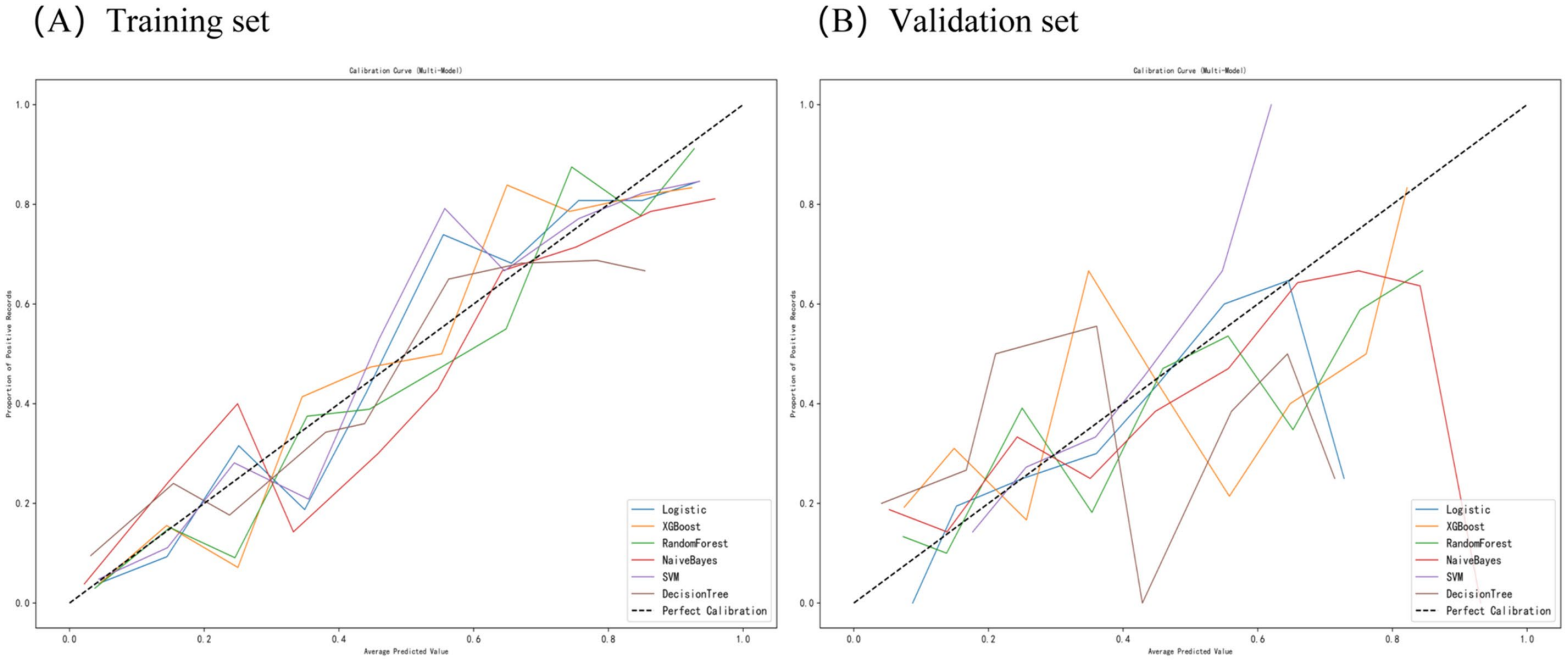
Calibration curves of prediction models for cognitive impairment in older adults with hearing loss.

**Figure 4:**
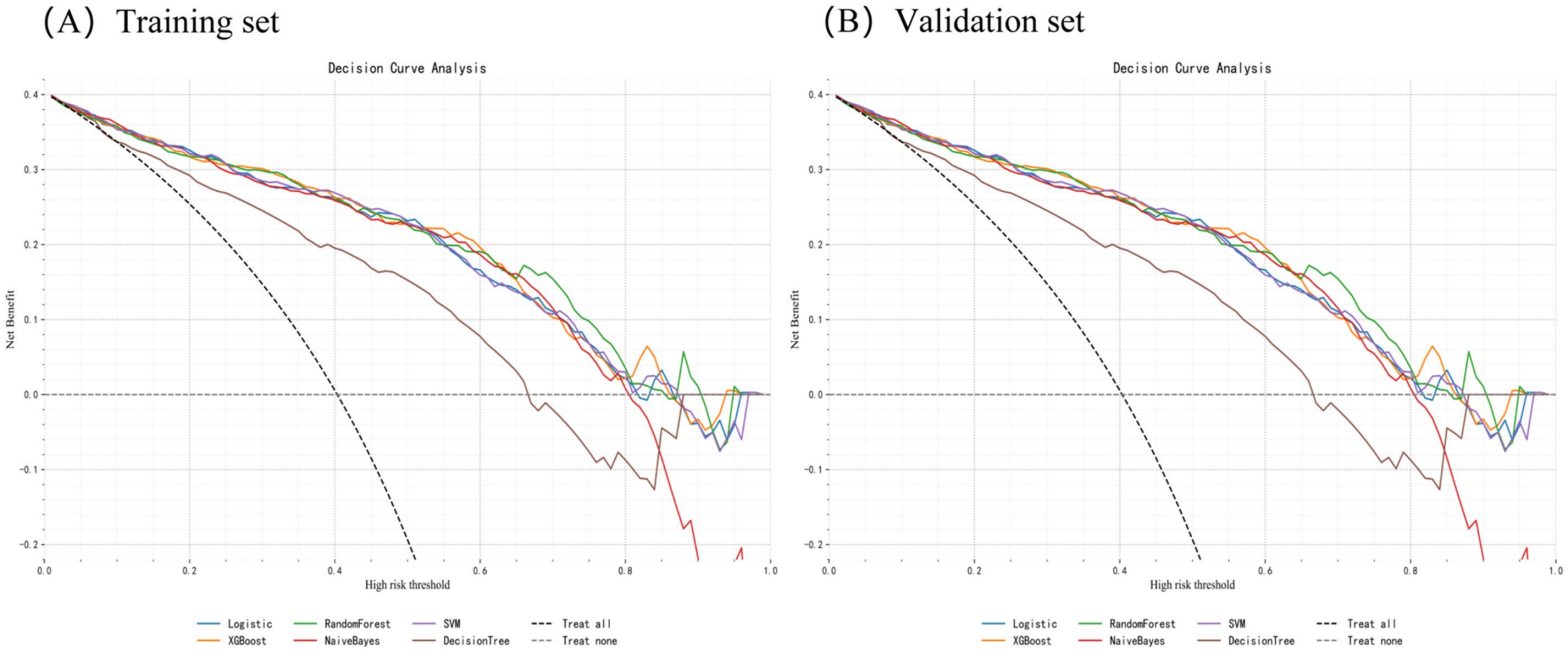
Decision curve analysis curves of prediction models for cognitive impairment in older adults with hearing loss.

Based on the five feature variables selected by LASSO regression, six commonly used machine-learning prediction models were developed, including Logistic regression, Decision Tree, Naive Bayes, Random Forest, SVM, and XGBoost. Model performance was compared in both the training set and the internal validation set using AUC, F1 score, specificity, sensitivity, Youden index, NPV, PPV, and DeLong’s test, with ROC, calibration, and decision curve analyses providing additional visual evaluation (Table 4; Figures 2–4). In the training set, XGBoost showed the best apparent performance, with the highest AUC (0.950), F1 score (0.867), specificity (0.908), sensitivity (0.869), Youden index (0.777), NPV (0.912), and PPV (0.866), while Random Forest also demonstrated favorable performance, with an AUC of 0.935 and an F1 score of 0.836. In the internal validation set, Random Forest showed the best overall predictive performance, achieving the highest AUC (0.850), F1 score (0.666), Youden index (0.484), and NPV (0.798), with a specificity of 0.862, sensitivity of 0.622, and PPV of 0.733. Although XGBoost ranked second in terms of AUC in the validation set (AUC = 0.817), its F1 score (0.523), sensitivity (0.430), and Youden index (0.333) were lower than those of Random Forest, suggesting relatively weaker classification performance for identifying cognitively impaired individuals. Compared with the Logistic regression model, Random Forest and XGBoost showed significantly better discrimination in both the training and internal validation sets (all *p* < 0.001). Considering its superior validation performance and better balance among discrimination, sensitivity, specificity, NPV, PPV, and generalizability, Random Forest was selected as the final candidate model for subsequent hyperparameter tuning and SHAP-based interpretability analysis.

**Table 4:** Performance comparison of machine learning–based prediction models for cognitive impairment in older adults with hearing loss.

Model name	AUC	F1	Specificity	Sensitivity	Youden index	NPV	PPV	*p* value
Training set								
Logistic	0.893	0.784	0.856	0.782	0.638	0.854	0.787	Reference
DecisionTree	0.850	0.730	0.810	0.739	0.550	0.825	0.731	0.004
NaiveBayes	0.892	0.793	0.818	0.834	0.652	0.880	0.756	0.222
Random Forest	0.935	0.836	0.887	0.839	0.726	0.891	0.835	<0.001
SVM	0.890	0.786	0.846	0.794	0.641	0.859	0.778	0.186
XGBoost	0.950	0.867	0.908	0.869	0.777	0.912	0.866	<0.001
Internal validation set								
Logistic	0.726	0.525	0.801	0.486	0.288	0.744	0.590	Reference
DecisionTree	0.712	0.524	0.754	0.533	0.287	0.730	0.566	0.997
NaiveBayes	0.735	0.601	0.736	0.625	0.361	0.772	0.584	0.870
Random Forest	0.850	0.666	0.862	0.622	0.484	0.798	0.733	<0.001
SVM	0.703	0.511	0.767	0.515	0.282	0.738	0.521	0.766
XGBoost	0.817	0.523	0.903	0.430	0.333	0.734	0.738	<0.001

### 3.6 Hyperparameter tuning of the final model

**Figure 5:**
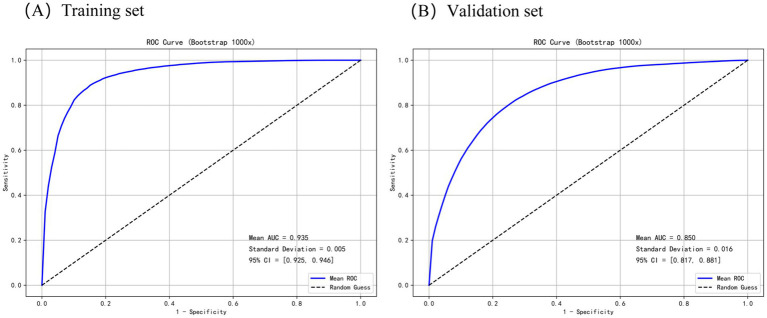
ROC curves of the Random Forest model before hyperparameter tuning in the training and validation sets.

**Figure 6:**
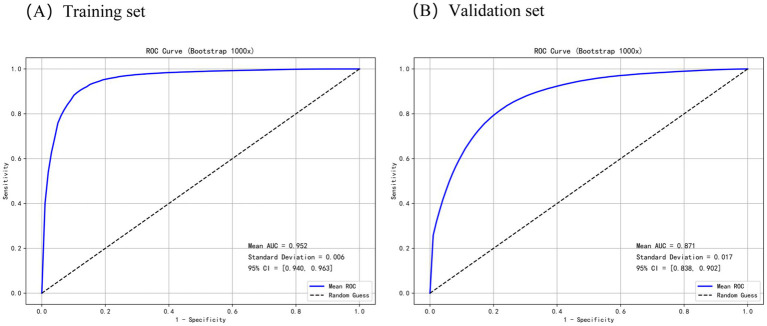
ROC curves of the Random Forest model after hyperparameter tuning in the training and validation sets.

Based on the preliminary comparison of machine learning models, the Random Forest model showed good overall predictive performance in the validation cohort and was therefore selected as the final candidate model for further optimization. To improve model stability and generalization ability, grid search combined with five-fold cross-validation was performed in the training cohort to optimize the key hyperparameters of the Random Forest model. The optimized Random Forest model consisted of 300 decision trees, with a maximum tree depth of 5, a maximum of 20 terminal leaf nodes, a minimum of 5 samples required for internal node splitting, and balanced class weights to reduce the influence of class imbalance on model performance. On this basis, Bootstrap resampling with 1,000 repetitions was further conducted to assess the robustness of the optimized model. As shown in Figure 5, before hyperparameter tuning, the Random Forest model achieved an AUC of 0.935 in the training cohort and 0.850 in the validation cohort. After hyperparameter tuning, the ROC curves shown in Figure 6 demonstrated improved discrimination, with AUC values of 0.952 and 0.871 in the training and validation cohorts, respectively. Detailed performance metrics before and after hyperparameter tuning are presented in Table 5. In the training cohort, the F1 score increased from 0.836 to 0.868, the sensitivity increased from 0.839 to 0.885, the specificity increased from 0.887 to 0.898, the Youden index increased from 0.726 to 0.783, the positive predictive value increased from 0.835 to 0.841, and the negative predictive value increased from 0.835 to 0.928. In the validation cohort, the F1 score increased from 0.666 to 0.737, the sensitivity increased from 0.622 to 0.779, the Youden index increased from 0.484 to 0.583, and the negative predictive value increased from 0.733 to 0.863. Although the specificity decreased from 0.862 to 0.804 and the positive predictive value decreased from 0.733 to 0.703 in the validation cohort after tuning, the overall discrimination and classification performance of the model improved, particularly in identifying patients with cognitive impairment and reducing false-negative predictions. Therefore, the optimized Random Forest model was selected as the final prediction model, and SHAP analysis was subsequently used for model interpretability.

**Table 5:** Performance of the Random Forest model before and after hyperparameter tuning.

Random Forest	AUC	F1	Specificity	Sensitivity	Youden index	PPV	NPV
Training-before	0.935	0.836	0.887	0.839	0.726	0.835	0.835
Training-after	0.952	0.868	0.898	0.885	0.783	0.841	0.928
Validation-before	0.850	0.666	0.862	0.622	0.484	0.733	0.733
Validation-after	0.871	0.737	0.804	0.779	0.583	0.703	0.863

### 3.7 SHAP analysis of the Random Forest model

**Figure 7:**
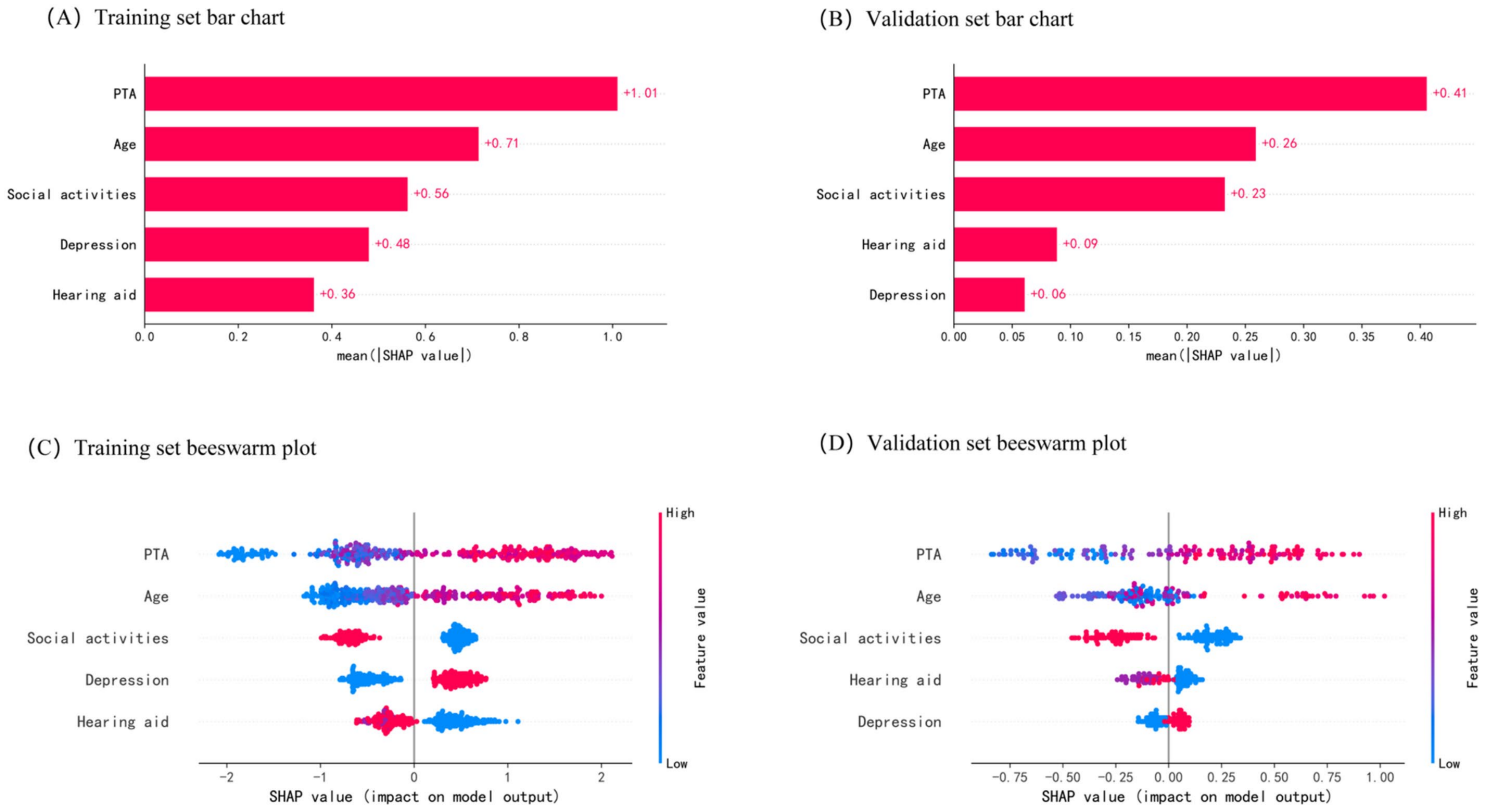
SHAP analysis of the optimized Random Forest prediction model for cognitive impairment in older adults with hearing loss.

SHAP analysis was performed to interpret the optimized Random Forest model after hyperparameter tuning (Figure 7). The SHAP feature-importance bar plot showed that, in the training set, the variables ranked by contribution from highest to lowest were PTA, age, social activities, depression, and hearing-aid use. In the validation set, the ranking was PTA, age, social activities, hearing-aid use, and depression. The SHAP beeswarm plots further demonstrated the direction of each predictor’s effect on model output. Higher PTA and older age were generally associated with positive SHA*p* values, indicating an increased predicted probability of cognitive impairment. Depression also tended to increase the predicted risk, although its contribution was smaller in the validation set. In contrast, participation in social activities was mainly associated with negative SHAP values, suggesting a protective effect against cognitive impairment. Hearing-aid use also contributed to model prediction and was generally associated with a reduced predicted risk. Overall, the SHAP results indicated that hearing severity, age, and social participation were the most influential factors in the optimized Random Forest model.

## 4 Discussion

In this study, the prevalence of cognitive impairment among older adults with hearing loss was as high as 40.8%, which is markedly higher than that reported in the general older population (20). Cognitive dysfunction substantially reduces quality of life in older adults and is often difficult to reverse once it occurs. Early identification of risk factors for cognitive impairment and timely intervention are of great importance for improving patients’ quality of life and slowing disease progression. Because the clinical manifestations of older adults with hearing loss are relatively complex, and traditional cognitive assessment methods may not identify cognitive impairment in a timely and effective manner, treatment and intervention can be delayed. Previous studies have shown that hearing loss not only directly affects communication ability in older adults but may also interact with other symptoms such as headache and dizziness/vertigo, thereby masking or delaying the early diagnosis of cognitive dysfunction (31). Therefore, developing a machine-learning prediction model to assist clinicians and healthcare providers in the early identification of high-risk patients has practical clinical significance.

The findings of this study are consistent with previous research, indicating that both age and the severity of hearing loss are important risk factors for cognitive impairment in older adults with hearing loss (32). With increasing age, physiological functions gradually decline, and the brain undergoes inevitable neurodegenerative changes. In particular, atrophy in brain regions related to memory, learning, and cognitive control—such as the hippocampus and prefrontal cortex—is closely associated with memory decline and impairments in attention and executive function (33, 34). Meanwhile, this study found a positive correlation between the degree of hearing loss and cognitive impairment; that is, the more severe the hearing loss, the higher the risk of cognitive dysfunction (35). A plausible explanation is that reduced hearing limits daily communication, decreases social interaction and participation, and reduces cognitive stimulation, thereby accelerating cognitive decline; we found that hearing loss was associated with reduced participation in social activities (36). However, the neurophysiological mechanisms and causal relationship between hearing loss and cognitive decline remain incompletely understood (37). Current leading hypotheses include the “cognitive reserve depletion” hypothesis, which posits that auditory processing requires greater attentional resources and competes with other cognitive tasks, potentially accelerating the long-term depletion of cognitive reserve (38), and the “sensory deprivation” hypothesis, which suggests that reduced auditory input leads to degeneration of auditory pathways and triggers an ascending cascade effect, whereby the brain may compensate at the expense of certain cognitive functions (39). Other studies have suggested that hearing decline is associated with changes in neurotransmitter levels in the auditory cortex and alterations in functional network connectivity, which may further affect cognitive performance (40). In addition, frailty may serve as an underlying contextual factor contributing to concurrent declines in hearing and cognition, making the association between the two more pronounced in some older populations (32). Future research should integrate neuroimaging, psychological assessments, and basic experimental evidence, and adopt more rigorous longitudinal or interventional designs to clarify key pathways and causal chains, thereby providing a basis for developing precise intervention strategies.

Unlike physiological factors, psychological factors are more amenable to intervention in the management of cognitive impairment among older adults with hearing loss (41). In this study, depression was identified as an important risk factor for cognitive impairment in this population. Hearing loss often restricts communication and increases feelings of social isolation, which in turn reduces social participation and may trigger anxiety or depression; negative emotions consume cognitive resources and may create a vicious cycle of “hearing decline–emotional problems–cognitive deterioration” (42). Previous studies have suggested that depression and cognitive impairment overlap to some extent in regions of structural brain damage, such as reduced gray matter volume in the temporal gyri and prefrontal cortex, and depression-related damage in these brain areas may also directly affect cognitive function (43–45). In addition, depression may activate the hypothalamic–pituitary–adrenal axis, inducing stress responses, neuroinflammation, and neurotransmitter imbalance, thereby further damaging brain regions involved in cognition and exacerbating cognitive impairment (46, 47). Therefore, health management should encourage older adults with hearing loss to actively participate in social activities to reduce the risk of depression, while strengthening health education for patients and their families. Community-based primary healthcare services should conduct screening and follow-up for hearing loss and depression, enabling early prevention, early detection, and early intervention. Future research could integrate multidisciplinary approaches such as neuroimaging, audiology, and neuropsychology to further elucidate the psychological–physiological mechanisms by which depression affects cognition, thereby providing evidence for precision interventions.

Lifestyle and behavioral factors are shaped by individual habits and are the most amenable to intervention. This study revealed an interesting finding: participation in social activities and hearing-aid use were associated with a lower risk of cognitive impairment among older adults with hearing loss. Previous studies have shown that social interaction involves complex cognitive processing such as language comprehension, reasoning, and memory, providing continuous stimulation to the brain and activating multiple brain regions, thereby helping to slow cognitive decline (48–50). However, our survey found that social participation in this population was generally low, mainly because hearing decline leads to communication difficulties, which in turn reduces social activities and increases feelings of detachment and loneliness; social disengagement and depression are both closely associated with cognitive decline (51). Therefore, increasing social participation has substantial intervention potential. Community programs and family support may encourage older adults to maintain social interaction, thereby alleviating loneliness and depression and promoting cognitive health (52). Nevertheless, this study assessed only whether participants engaged in social activities; different forms of social engagement (e.g., visiting relatives and friends, attending senior universities) may have differential effects on cognition. Future studies should further stratify activity types and frequencies to develop more individualized recommendations (53). In addition, hearing-aid use emerged as a protective factor in this study, an important finding, although the literature remains mixed (54). Potential mechanisms include improved auditory input, reduced cognitive load required for auditory processing, and enhanced social interaction, thereby indirectly benefiting cognition (55, 56). However, the effect is highly dependent on actual use. Many older adults wear hearing aids inconsistently due to discomfort, operational difficulty, forgetfulness, or stigma, which may contribute to discrepancies across studies (57). Future work should focus on promoting sustained use, improving comfort and usability, and reducing psychological barriers through individualized fitting and counseling, in order to more fully realize the potential cognitive protective effects of hearing aids.

In terms of model development, this study adopted the IFPHRM as the theoretical framework. Based on multidimensional influencing factors, LASSO regression was used to identify five core predictors—age, PTA, depression, hearing-aid use, and social activities—and the predictive performance of six commonly used machine-learning algorithms was further compared. Although XGBoost achieved the best apparent performance in the training set, Random Forest demonstrated the best overall predictive performance in the internal validation set, with higher AUC, F1 score, Youden index, and NPV, suggesting better discrimination, classification performance, and generalizability. In contrast, the performance of XGBoost declined in the internal validation set despite its strong performance in the training set, indicating relatively limited generalization ability. Therefore, Random Forest was selected as the final candidate model for further hyperparameter tuning. After grid-search optimization combined with five-fold cross-validation, the optimized Random Forest model showed further improvement in the validation cohort, particularly in AUC, F1 score, sensitivity, Youden index, and NPV. Combined with the ROC, calibration, and DCA curves, the optimized Random Forest model was ultimately selected as the final prediction model in this study. SHAP-based interpretability analysis further supported the robustness of the selected predictors. These findings were generally consistent with the multivariable analysis, indicating that cognitive impairment in older adults with hearing loss is not driven by a single factor but rather results from the combined effects of hearing deterioration, aging, emotional problems, and behavioral adaptation.

From a public health and clinical translation perspective, the optimized model has practical value because it incorporates five low-cost and readily available indicators: age, PTA, depression, hearing-aid use, and social activities. These variables can be obtained in routine otolaryngology visits, community hearing screening, chronic disease management, and health examinations for older adults, which enhances the feasibility of model implementation in real-world settings. Rather than replacing formal cognitive assessment, this model may serve as an auxiliary risk-stratification tool to help clinicians and community healthcare providers identify older adults with hearing loss who require further cognitive evaluation, psychological support, hearing rehabilitation, or targeted follow-up. In resource-limited primary care settings, such a tool may help prioritize high-risk individuals, support individualized monitoring, and improve the efficiency of preventive interventions. Therefore, the model may facilitate a shift from passive identification of cognitive impairment to proactive early warning and targeted management among older adults with hearing loss. Nevertheless, this study has several limitations. First, because this was a cross-sectional study, temporal causality between the identified predictors and cognitive impairment could not be established. Second, the sample was drawn from healthcare institutions in a single region and related screening settings; although temporal split validation was performed, the model’s generalizability still needs to be further verified in larger samples, multicenter studies, and populations from different regions.

## 5 Conclusion

This study found a high prevalence of cognitive impairment among older adults with hearing loss. Age, the severity of hearing loss, and depression were major risk factors, whereas hearing-aid use and participation in social activities appeared to be protective factors. The optimized Random Forest prediction model based on these five indicators demonstrated improved discrimination and clinical utility after hyperparameter tuning. Rather than serving as a standalone diagnostic tool, this model may be more appropriately used as a low-cost auxiliary risk-stratification tool to identify older adults with hearing loss who require further cognitive assessment and targeted intervention in clinical and community settings.

## Data Availability

The original contributions presented in the study are included in the article/supplementary material, further inquiries can be directed to the corresponding author/s.
